# Supplementary material for: Ambient Air Pollution and Sudden Infant Death Syndrome in Korea: A Time-Stratified Case-Crossover Study
Source: Int J Environ Res Public Health. 2019 Sep 6;16(18):3273. doi: 10.3390/ijerph16183273 (PMC6765778; doi:10.3390/ijerph16183273)
Supplement: Supplementary file 1 [file ijerph-16-03273-s001.pdf]

Supplement

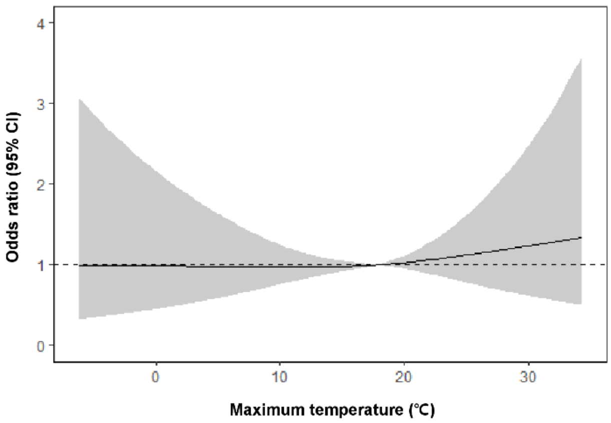

Figure S1. Association between daily maximum temperature and odds ratio of SIDS, Republic of Korea, 2009-2013. Adjusting for daily average humidity. CI, confidence interval.

Table S1. Estimated effects of SIDS per interquartile range increment of ambient air pollutants stratified the range of maximum daily temperature.

	Estimates (95% CI) ⁺			
**Temperature**	**<18℃**			
**Lag (days)**	**PM_{10}**	**NO_2**	**CO**	**SO_2**
Lag 0	0.67 (0.34-1.29)	0.78 (0.29-2.10)	0.47 (0.19-1.13)	1.13 (0.55-2.32)
Lag 1	0.93 (0.47-1.83)	0.56 (0.19-1.70)	0.83 (0.34-2.03)	0.80 (0.39-1.62)
Lag 2	1.02 (0.62-1.69)	0.60 (0.21-1.77)	0.90 (0.42-1.90)	0.82 (0.42-1.60)
Lag 3	0.91 (0.53-1.57)	0.48 (0.18-1.30)	0.76 (0.38-1.52)	1.04 (0.53-2.07)
MA0-3	0.83 (0.39-1.77)	0.48 (0.11-2.07)	0.53 (0.18-1.56)	0.79 (0.31-2.05)
	**18℃ – 29℃**			
Lag 0	1.04 (0.89-1.20)	1.12 (0.86-1.45)	1.11 (0.94-1.32)	1.12 (0.94-1.33)
Lag 1	1.05 (0.91-1.22)	1.12 (0.88-1.44)	1.15 (0.98-1.36)	1.07 (0.90-1.28)
Lag 2	1.15 (1.01-1.31)*	1.21 (0.94-1.55)	1.16 (0.97-1.38)	1.03 (0.84-1.25)
Lag 3	1.09 (0.96-1.24)	1.25 (0.97-1.60)	1.19 (1.01-1.39)*	1.01 (0.83-1.24)
MA0-3	1.16 (0.97-1.40)	1.25 (0.90-1.73)	1.25 (1.01-1.54)*	1.16 (0.92-1.48)
	**≥29℃**			
Lag 0	0.97 (0.76-1.24)	1.63 (1.01-2.66)*	1.50 (0.93-2.42)	0.90 (0.60-1.35)
Lag 1	1.01 (0.83-1.23)	1.52 (0.94-2.47)	1.73 (1.11-2.70)*	1.32 (0.92-1.90)
Lag 2	1.09 (0.90-1.32)	0.93 (0.58-1.49)	1.20 (0.78-1.85)	0.95 (0.67-1.33)
Lag 3	1.07 (0.95-1.21)	1.03 (0.64-1.65)	1.31 (0.85-2.04)	1.12 (0.76-1.64)
MA0-3	1.00 (0.77-1.31)	1.31 (0.69-2.52)	1.65 (0.93-2.93)	0.95 (0.57-1.57)

*Statistically significant values ($p<0.05$). ⁺: adjusting for daily average temperature and humidity. Estimated effects were expressed as odds ratio per 27.8 μg/m^3 increment in PM_{10} level, 15.7 ppb increment in NO_2 level, 215.8 ppb increment in CO level, and 2.8 ppb increment in SO_2 level. CI, confidence interval; PM_{10}, particulate matter less than 10 μm in diameter; NO_2, nitrogen dioxide; ppb, parts per billion; CO, carbon monoxide; SO_2, sulfur dioxide; MA0-3, moving average of 0 to 3 days.
